# Supplementary material for: Inhibition of BKCa channels protects neonatal hearts against myocardial ischemia and reperfusion injury
Source: Cell Death Discov. 2022 Apr 7;8:175. doi: 10.1038/s41420-022-00980-z (PMC8989942; doi:10.1038/s41420-022-00980-z)
Supplement: Supplementary file 1 — Supplementary material [file 41420_2022_980_MOESM1_ESM.docx]

**Inhibition of BK_Ca_ channels protects neonatal hearts against myocardial ischemia and reperfusion injury**

Shridhar Sanghvi^1,2^, Kalina Szteyn^1^, Devasena Ponnalagu^1^, Divya Sridharan^3^, Alexander Lam^4^, Inderjot Hansra^1^, Ankur Chaudhury^4^, Uddalak Majumdar^5^, Andrew R. Kohut^4,6^, Shubha Gururaja Rao^7^, Mahmood Khan^1,3^, Vidu Garg^5,8^, Harpreet Singh^1,2,4^

^1^Department of Physiology and Cell Biology, The Ohio State University Wexner Medical Center, Columbus, OH.

^2^ Department of Molecular Cellular and Developmental Biology, The Ohio State University, Columbus, OH.

^3^Department of Emergency Medicine, Wexner Medical Center, The Ohio State University, Columbus, OH.

^4^Division of Cardiology, Department of Medicine, Drexel University College of Medicine, Philadelphia, PA.

^5^Center for Cardiovascular Research and The Heart Center, Nationwide Children's Hospital, Columbus, OH.

^6^Division of Cardiology, Department of Medicine, Perelman School of Medicine, University of Pennsylvania, Philadelphia, PA.

^7^Department of Pharmaceutical and Biomedical Sciences, The Raabe College of Pharmacy, Ohio Northern University, Ada, OH.

^8^Department of Pediatrics, The Ohio State University, Columbus, Ohio

**Corresponding author: Harpreet.singh@osumc.edu**

**Preprint available at** [**https://www.biorxiv.org/content/10.1101/2021.11.02.466585v1**](https://www.biorxiv.org/content/10.1101/2021.11.02.466585v1)

**Supplementary Figure 1**

Validation of BK_Ca_ antibody in cardiomyocytes isolated from adult *Kcnma1^+/+^* and *Kcnma1^-/-^* mice. Dynamin 1 is used as a loading control. Molecular weights are given in KDa.


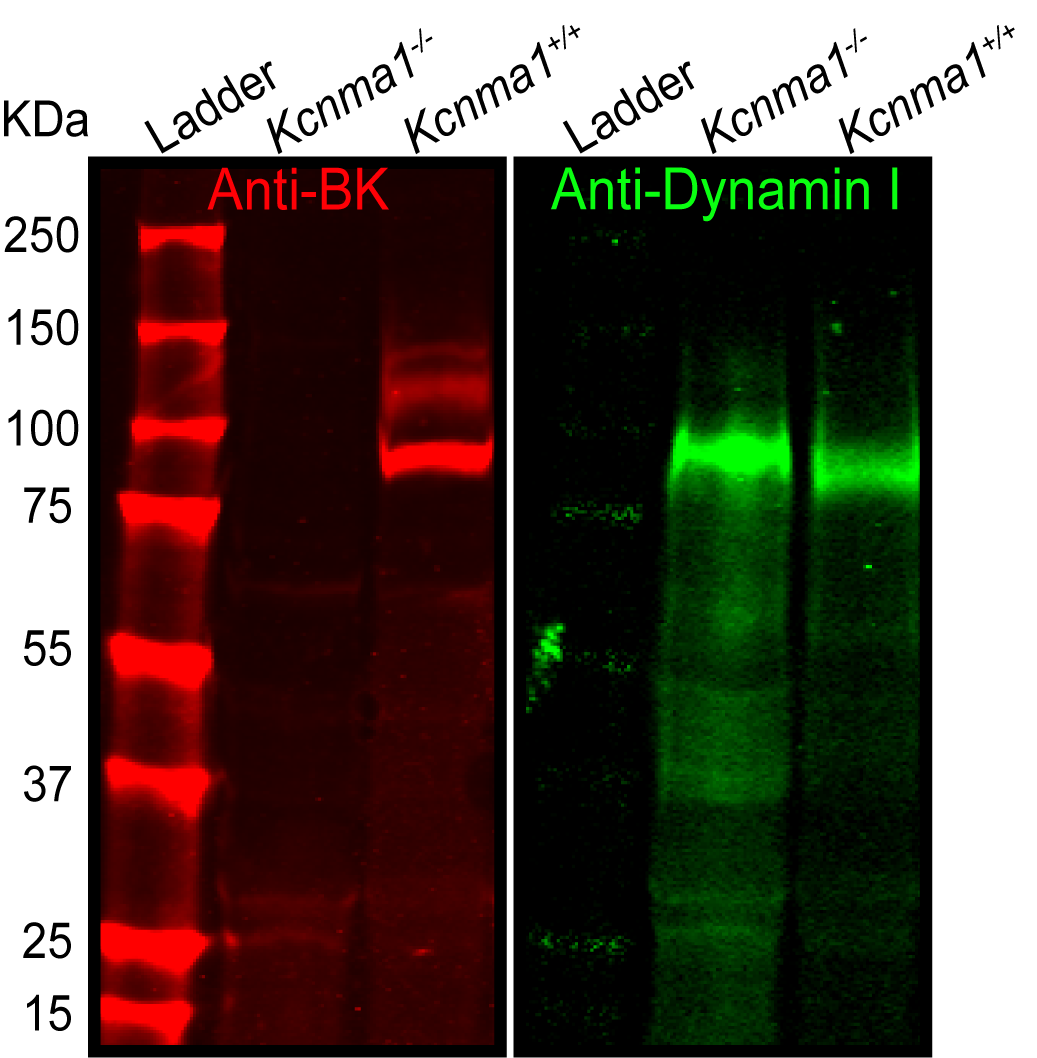


**Supplementary Figure 2.**

**
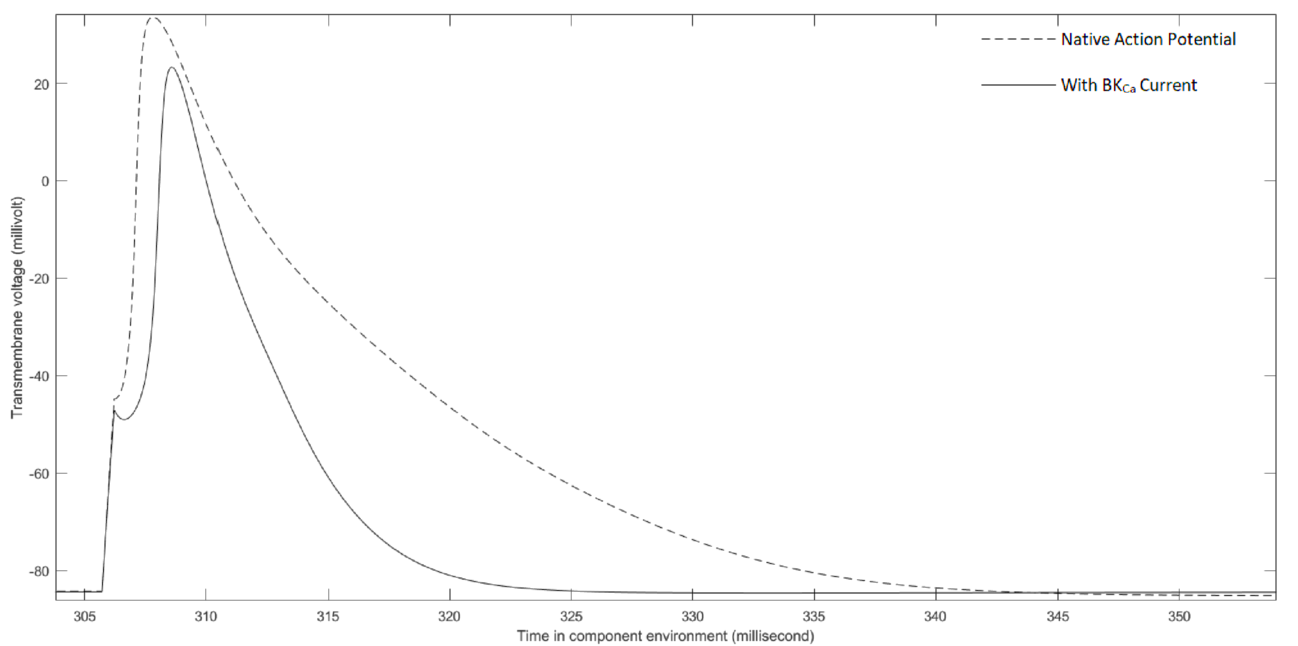
Alteration of adult murine ventricular cardiomyocyte AP with the integration of BK_Ca_ channel into the cell membrane**. As compared to the native action potential (dotted line), integration of the BK_Ca_ current into the murine ventricular AP (solid line) causes an early notch in the action potential, decreased maximum depolarization amplitude, and more rapid repolarization which decreases the duration of the action potential.

**Supplementary Table 1. Primers for qPCR.**

| **Splice variant** | **Primer** | **Sequence** | **Size (bp)** |
| --- | --- | --- | --- |
| Total | Forward primer | 5’ CCATTAAGTCGGGCTGATTTAAG 3′ | 187 |
|  | Reverse primer | 5′ CCTTGGGAATTAGCCTGCAAGA 3′ |  |
| BK_Ca_-DEC | Forward primer | 5′ GGTTTACAGATGAGCCGGATA 3′ | 134 |
|  | Reverse primer | 5′ CATCTTCAACTTCTCTGATTGG 3′ |  |
| GAPDH | Forward primer | 5’ ACAGCAACAGGGTGGTGGA 3’ | 117 |
|  | Reverse primer | 5’ TTTGAGGGTGCAGCGAACTT 3’ |  |
