# Supplementary figures and images for: Inhibition of BKCa channels protects neonatal hearts against myocardial ischemia and reperfusion injury
Source: Cell Death Discov. 2022 Apr 7;8:175. doi: 10.1038/s41420-022-00980-z (PMC8989942; doi:10.1038/s41420-022-00980-z)

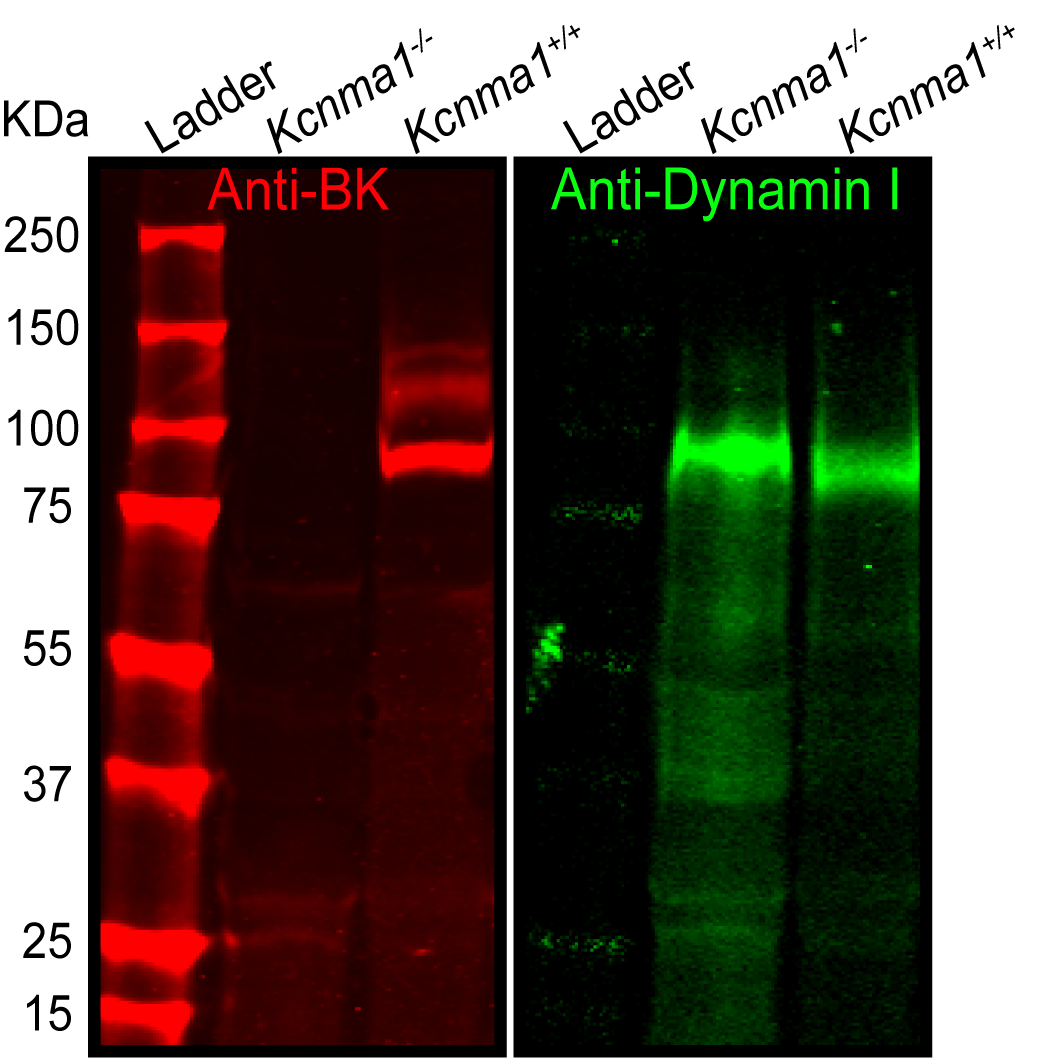

Supplement: Supplementary file 2 — Supplementary Figure 1 [file 41420_2022_980_MOESM2_ESM.tif]

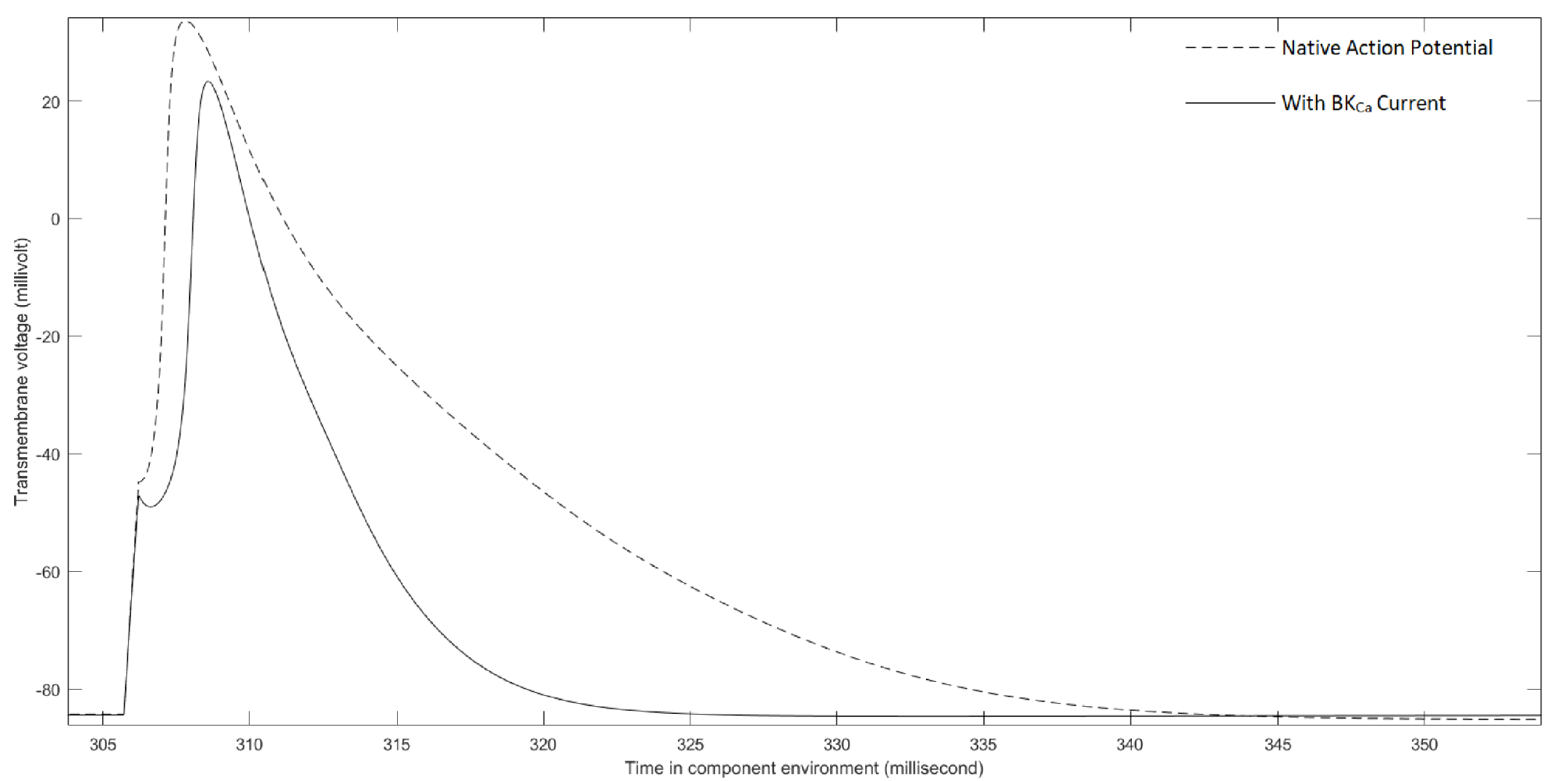

Supplement: Supplementary file 3 — Supplementary Figure 2 [file 41420_2022_980_MOESM3_ESM.tif]
